# Effects of epinephrine administration in out-of-hospital cardiac arrest based on a propensity analysis

**DOI:** 10.1186/2052-0492-1-12

**Published:** 2013-12-04

**Authors:** Mineji Hayakawa, Satoshi Gando, Hirotoshi Mizuno, Yasufumi Asai, Yasuo Shichinohe, Isao Takahashi, Hiroshi Makise

**Affiliations:** Emergency and Critical Care Center, Hokkaido University Hospital, N14W5, Kita-ku, Sapporo, 060-8648 Japan; Department of Traumatology and Critical Care Medicine, Sapporo Medical University Hospital, Sapporo, Japan; Department of Emergency and Critical Care Medicine, National hospital organization Hokkaido Medical Center, Sapporo, Japan; Emergency Department, Teine Keijinkai Hospital, Sapporo, Japan; Emergency and Critical Care Center, Sapporo City General Hospital, Sapporo, Japan

**Keywords:** Cardiac arrest, Epinephrine, Prehospital, Propensity analysis, Utstein

## Abstract

**Background:**

Epinephrine administration has been advocated for cardiopulmonary resuscitation (CPR) for decades. Despite the fact that epinephrine administration during CPR is internationally accepted, the effects of the prehospital epinephrine administration still remain controversial. We investigated the effects of epinephrine administration on patients with out-of-hospital cardiac arrest based on a propensity analysis with regard to the ‘CPR time’.

**Methods:**

From April 1, 2007, to December 31, 2009, 633 out-of-hospital cardiac arrest patients with bystander witnesses were included in the present study. To rule out any survival bias, we used the propensity scores, which included CPR time. CPR time was defined as the time span from when the emergency medical technicians started CPR until either the return of spontaneous circulation or arrival at the hospital. After performing propensity score matching, the epinephrine and no-drug groups each included 141 patients. The primary study endpoint was a favorable neurological outcome at 30 days after cardiac arrest.

**Results:**

After propensity score matching, the frequency of the return of spontaneous circulation before arrival at the hospital in the matched epinephrine group was higher than that in the matched no-drug group (27% vs. 13%, *P* = 0.002). However, the frequency of a favorable neurological state did not differ between the two groups. With regard to the frequency of a favorable neurological state in the patients, the adjusted odds ratio of the time span from cardiac arrest to the first epinephrine administration was 0.917 (95% confidence interval 0.850–0.988, *P* = 0.023) per minute.

**Conclusions:**

In patients with witnessed out-of-hospital cardiac arrest, prehospital epinephrine administration was associated with increase of the return of spontaneous circulation before arrival at the hospital. Moreover, the early administration of epinephrine might improve the overall neurological outcome.

## Background

Epinephrine administration has been advocated for cardiopulmonary resuscitation (CPR) for decades and is still included in new recommendations [1]. Several previous reports have indicated that administration of epinephrine increased the frequency of the return of spontaneous circulation (ROSC) [2–6].

Recently, a large observational propensity analysis of prehospital epinephrine administration was reported based on a nationwide Utstein database in Japan [7]. They indicated that the administration of epinephrine was associated with a deterioration of the neurological outcome of patients with out-of-hospital cardiac arrest, although the frequency of ROSC did increase [7]. However, their report was considered to have an important bias. For instance, some patients did not require epinephrine because of early ROSC, and these patients tended to have a better neurological outcome [8, 9]. Other patients had a large chance to be administrated epinephrine because of long time span from starting CPR until ROSC, and these patients tend to have a worse neurological outcome [8, 9]. The chance to perform prehospital epinephrine administration for patients with out-of-hospital cardiac arrest is limited to the time span from the moment that the emergency medical technicians (EMTs) started CPR until either ROSC or arrival at the hospital. We defined this time span as ‘CPR time’ in the present study. Previously, Ong et al. indicated that CPR time is what truly leads to an important bias in a study comparing CPR with and without epinephrine for out-of-hospital cardiac arrest [8].

The most recent randomized double-blind placebo-control trial of the use of epinephrine in out-of-hospital cardiac arrest patients was reported by Jacobs et al. [6]. They indicated that the use of epinephrine increased the short-term survival rate in out-of-hospital cardiac arrest patients [6]. However, the neurological outcome was not improved in that randomized control trial, as same as a previous randomized control trial [2, 6]. There was no potential bias in the randomized control trial. However, it is likely that the administration of epinephrine did not improve the neurological outcome in that study because the two studies included cardiac arrest patients without bystander witness [2, 6].

In the present study, we used propensity analyses that included CPR time and investigated the effects of epinephrine administration before arrival at the hospital for patients with witnessed out-of-hospital cardiac arrest. In particular, the effects of early administration of epinephrine on the neurological outcome in out-of-hospital cardiac arrest patients were investigated.

## Methods

The present retrospective study was approved by the institutional review committee of Hokkaido University Hospital.

### Patients

Sapporo city started monitoring out-of-hospital cardiac arrests according to the Utstein template [10] on April 1, 2002. In Sapporo, EMTs have provided advanced life support according to the international Guidelines 2005 [11] since April 1, 2007. The present study retrospectively analyzed the Utstein template [10] records from April 1, 2007, to December 31, 2009. The patients who had out-of-hospital cardiac arrest with bystander witnesses were included in the study. Patients who had cardiac arrest caused by non-cardiac disease, and who already had their spontaneous circulation restored before arrival of EMTs were excluded from the study. Patients under 8 years of age were also excluded because EMTs were not permitted to administer epinephrine to these patients. Cardiac arrest was defined as the cessation of cardiac mechanical activity, which was manifested as unresponsiveness, apnea, and the absence of a pulse. All events were measured by the dispatch center or the automated defibrillator clock and recorded.

### Treatment procedures

Each patient was managed by an ambulance with three EMTs. In Sapporo, all EMT teams were permitted to use advanced airway devices and administer epinephrine when performing CPR during the study period. When cardiac arrest was detected in the patient, chest compressions and ventilation by a bag valve mask were immediately started by two EMTs. CPR was provided according to the international guidelines 2005 [11]. The other EMTs inserted an advanced airway device (esophageal obstructive airway, laryngeal mask airway, or tracheal tube). In Japan, tracheal tube was rarely used for cardiac arrest patients because only some EMTs who trained are permitted to use a tracheal tube. The EMTs applied an automated defibrillator if necessary. EMTs tried to gain peripheral venous access and administer 1 mg of epinephrine intravenously. Epinephrine was administrated intravenously every 4 min until ROSC or until arrival at the hospital. No other drug is permitted for use by EMTs in Japan. After the attempted defibrillation, insertion of an advanced airway device, and administration of epinephrine, the EMTs transferred the patients to a hospital while performing CPR. When EMTs were unable to gain peripheral venous access at the scene, they again tried to gain peripheral venous access in the ambulance after departure from the scene. The time span from when the EMTs started CPR until either ROSC or arrival at the hospital was defined CPR time. According to whether the patients received epinephrine out of the hospital, the patients were divided into an epinephrine group or a no-drug group. The patients in the no-drug group had not received epinephrine out-of-the-hospital because EMTs could not gain peripheral venous access until ROSC or until arrival at the hospital. After arrival at the hospital, all patients in both groups were provided advanced life support, including the administration of epinephrine.

### Outcome investigation

The study primary endpoint was a favorable neurological outcome 30 days after cardiac arrest. A favorable neurological outcome was defined as a cerebral performance category score of 1 (good performance) or 2 (moderate disability) [10]. The secondary endpoints were return of spontaneous circulation before arrival at the hospital, admission to the intensive care unit, and survival of 7 and 30 days after the cardiac arrest. The cerebral performance category score was assessed either directly or via a telephone interview by the physicians at each hospital.

### Statistical analysis

The SPSS 15.0 J statistical software package (SPSS Inc., Chicago, IL, USA) was used for all statistical analyses. Comparisons between the groups were made using unpaired Student’s *t* tests and the Chi-square test. A multiple logistic-regression model was used to carry out the propensity score evaluation. The propensity score was the conditional probability of receiving epinephrine administration out of the hospital. The observational variables without multicollinearity were combined into a multiple logistic-regression model. The predicted probability derived from the logistic equation was used as the propensity score for each patient. Patients in the 25th–75th percentile of propensity scores were selected before propensity score matching. Propensity score matching was automatically performed by the SPSS Propensity Matching Program developed by Painter (http://www.unc.edu/~painter/). Patients with differences in their propensity scores of less than 0.03 in each group were selected. After the propensity score matching, comparisons between the matched groups were made using paired Student’s *t* tests and the McNemar test. To investigate the relationship between early administration of epinephrine and outcomes, the odds ratios of the time span from cardiac arrest to the first epinephrine administration for primary and secondary outcomes were calculated by a multiple logistic-regression analysis.

A *P* value of <0.05 was considered to be statistically significant. Unless otherwise indicated, all data were expressed as the means ± SD.

## Results and discussion

### Results

**Figure 1:**
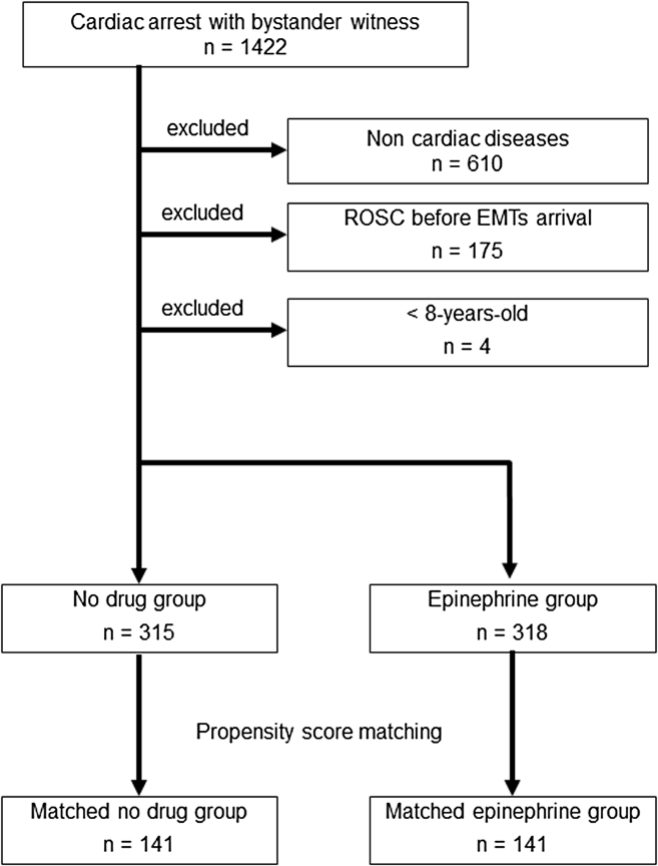
**A flow chart showing the inclusions and exclusions from the study.** Patients under 8 years of age were excluded from the present study because emergency medical technicians were not permitted to administer epinephrine to these patients.

During the study period, 1,422 patients with witnessed out-of-hospital cardiac arrest were transferred to the hospital by EMTs. A total of 789 patients were excluded from the present study (610 patients with non-cardiac disease, 175 patients with ROSC before EMTs arrival, and 4 patients less than 8 years old). The epinephrine group included 318 patients who could receive epinephrine outside of the hospital. The no-drug group included 315 patients who could not receive any drugs prior to arrival at the hospital (Figure 1). The characteristics of the patients in the two groups are presented in Table 1. No patients were used a tracheal tube in the both groups. In the no-drug group, the time from the start of CPR to ROSC was earlier than that in the epinephrine group (11.9 ± 8.1 min vs. 19.6 ± 6.9 min, *P* < 0.001).

**Table 1:** **Characteristics of the patients before propensity score matching**

	No drug	Epinephrine	***P*** value
	(***n*** = 315)	(***n*** = 318)	
Age (year)	71 ± 17	71 ± 16	0.887
Gender, male (%)	194 (62)	228 (72)	0.009
Bystander performed CPR, *n* (%)	141 (45)	140 (44)	0.873
VF/VT as initial rhythm at starting CPR by EMTs, *n* (%)	83 (26)	90 (28)	0.594
Asystole, *n* (%)	138 (44)	151 (48)	0.228
Pulseless electrical activity, *n* (%)	94 (30)	77 (24)	
VT, *n* (%)	2 (0.6)	1 (0.3)	
VF, *n* (%)	81 (26)	89 (28)	
Advanced life support by a physician in the ambulance, *n* (%)	165 (52)	167 (53)	1.000
From call receipt to ambulance stop (min)	6.3 ± 2.6	6.6 ± 2.8	0.141
From call receipt to start CPR by EMTs (min)	8.1 ± 3.8	8.1 ± 3.1	0.784
From witnessed cardiac arrest to start CPR by EMTs (min)	9.5 ± 7.6	9.7 ± 7.8	0.701
From the start of CPR by EMTs to departure from the scene (min)	13.3 ± 4.8	14.7 ± 4.3	<0.001
From the start of CPR by EMTs to arrival at the hospital (min)	25.2 ± 8.5	29.1 ± 8.4	<0.001
CPR time (min)	21.1 ± 9.6	26.9 ± 9.0	<0.001
From witnessed cardiac arrest to the first epinephrine administration (min)	-	22.5 ± 8.4	-
From start CPR by EMTs to the first epinephrine administration (min)	-	13.3 ± 5.9	-
Frequency of epinephrine administration until arrival at the hospital	-	2.5 ± 1.5	-
Outcome			
ROSC before arrival at the hospital, *n* (%)	78 (25)	73 (23)	0.641
From start CPR by EMTs to ROSC (min)	11.9 ± 8.1	19.6 ± 6.9	<0.001
Admission to the intensive care units, *n* (%)	126 (40)	124 (39)	0.808
Survival 7 days after the cardiac arrest, *n* (%)	86 (27)	65 (20)	0.050
Survival 30 days after the cardiac arrest, *n* (%)	76 (24)	53 (17)	0.023
Favorable neurological state 30 days after, *n* (%)	54 (17)	25 (8)	<0.001
Good performance	44 (14)	18 (6)	
Moderate disability	10 (3)	7 (2)	
Severe disability	4 (1)	5 (2)	0.009
Vagitative	18 (6)	23 (7)	
Dead	239 (76)	265 (93)	

Data are the mean ± SD or number (%) where appropriate. For comparison, unpaired Student’s *t* test or the Chi-square test were used when appropriate. *n*, number; CPR, cardiopulmonary resuscitation; EMT, emergency medical technician; VF, ventricular fibrillation; VT, ventricular tachycardia; ROSC, return of spontaneous circulation.

#### Propensity score matching between the epinephrine group and the no drug group

**Figure 2:**
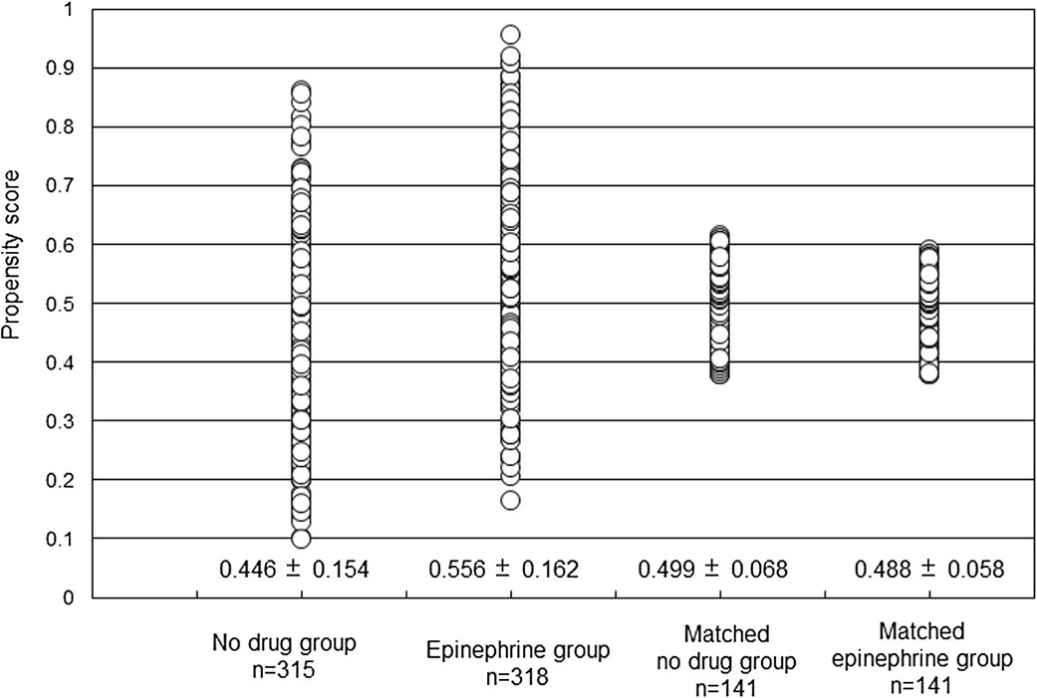
**Scatter diagram of propensity scores of conditional probability of receiving epinephrine outside of hospital.** The left side of the diagram shows the propensity scores in the two groups before matching. The right side of the diagram shows the propensity scores in the two groups after matching.

The following variables were combined into a multiple logistic regression model: age, gender, CPR performed by a bystander, VF/VT as the initial rhythm at starting CPR by EMTs, advanced life support by a physician in the ambulance, the time span from call receipt to start of CPR by EMTs, the duration from witnessed cardiac arrest to the start of CPR by EMTs, and CPR time. The following variables were excluded because of multicollinearity: the time span from call receipt to ambulance stop (vs. the time span from call receipt to start of CPR by EMTs), the time span from the start of CPR by EMTs to departure from the scene (vs. CPR time), and the time span from the start of CPR by EMTs to arrival at the hospital (vs. CPR time). When the multicollinearity was observed in the variables, more important variables were combined into a multiple logistic regression model. The predicted probability derived from the logistic equation was used as the propensity score for each patient. The propensity scores for the conditional probability of receiving epinephrine outside of the hospital were calculated for the two groups. A scatter diagram of the propensity scores before and after performing propensity score matching is presented in Figure 2. The characteristics of the patients in each group after the propensity score matching are presented in Table 2. The characteristics were not significantly different between the two groups, except the frequency of bystander performed CPR. While the frequency of ROSC in the matched epinephrine group was higher than that in the matched no-drug group (27% vs. 13%, *P* = 0.002), the other outcomes were not improved.

**Table 2:** **Characteristics of patients after propensity score matching**

	No drug	Epinephrine	***P*** value
	(***n*** = 141)	(***n*** = 141)	
Age (year)	72 ± 13	72 ± 18	0.752
Gender, male (%)	103 (73)	92 (65)	0.200
Bystander performed CPR, *n* (%)	54 (38)	71 (50)	0.044
Advanced life support by a physician in the ambulance, *n* (%)	72 (51)	68 (48)	0.694
VF/VT as initial rhythm at starting CPR by EMTs, *n* (%)	28 (20)	27 (19)	1.000
From call receipt to ambulance stop (min)	6.2 ± 2.5	6.4 ± 2.5	0.527
From call receipt to start CPR by EMTs (min)	7.9 ± 3.0	7.8 ± 3.1	0.845
From witnessed cardiac arrest to start CPR by EMTs (min)	10.1 ± 8.4	9.3 ± 6.9	0.377
From the start of CPR by EMTs to departure from the scene (min)	13.5 ± 4.3	13.6 ± 3.9	0.878
From the start of CPR by EMTs to arrival at the hospital (min)	24.4 ± 5.8	25.3 ± 5.6	0.162
CPR time (min)	23.3 ± 5.1	23.2 ± 4.6	0.977
From witnessed cardiac arrest to the first epinephrine administration (min)	-	21.8 ± 8.2	-
From start CPR by EMTs to the first epinephrine administration (min)	-	12.7 ± 5.0	-
Frequency of epinephrine administrations until arrival at the hospital	-	2.1 ± 1.0	-
Outcome			
ROSC before arrival at the hospital, *n* (%)	18 (13)	38 (27)	0.002
From start CPR by EMTs to ROSC (min)	20.3 ± 5.8	21.5 ± 4.7	-
Admission to the intensive care units, *n* (%)	44 (31)	57 (40)	0.112
Survival 7 days after the cardiac arrest, *n* (%)	22 (16)	28 (20)	0.429
Survival 30 days after the cardiac arrest, *n* (%)	19 (13)	22 (16)	0.728
Favorable neurological state 30 days after, *n* (%)	11 (8)	12 (9)	1.000

Data are the mean ± SD or number (%) where appropriate. For comparison, paired Student’s *t* test or the McNemar test was used when appropriate. *n*, number; CPR, cardiopulmonary resuscitation; EMT, emergency medical technician; VF, ventricular fibrillation; VT, ventricular tachycardia; ROSC, return of spontaneous circulation.

#### Effects of early administration of epinephrine in the epinephrine group

**Figure 3:**
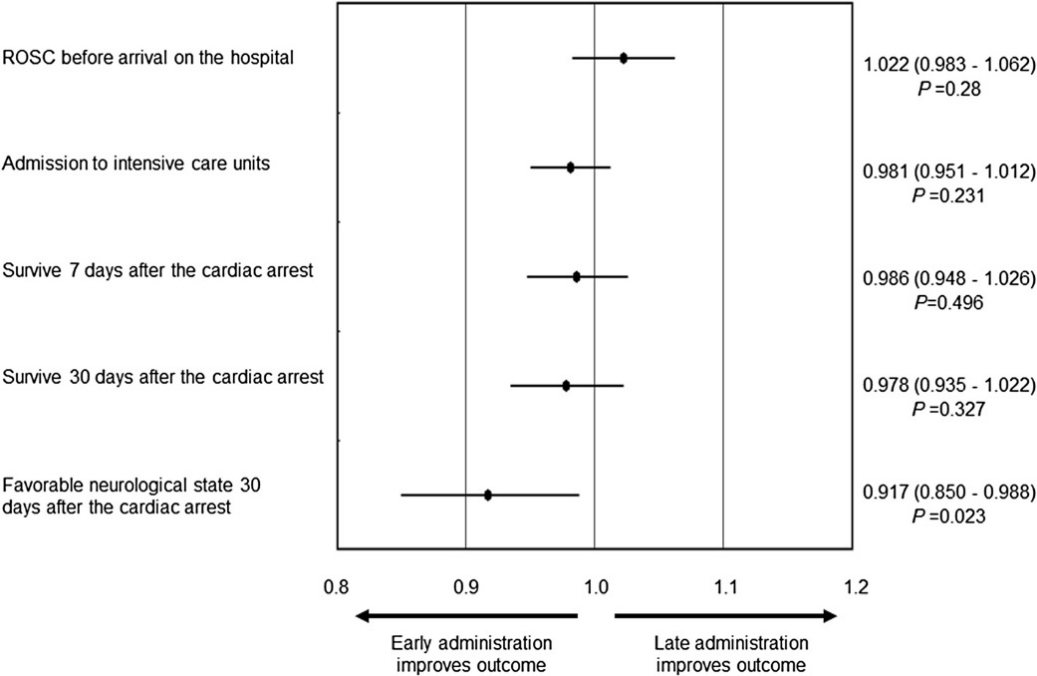
**Odds ratios of time span from cardiac arrest to first administration of epinephrine adjusted by CPR time.** Early administration of epinephrine increased the frequency of a favorable neurological outcome at 30 days after cardiac arrest, although it did not improve the other outcomes. The values in the right side of the figure were the odds ratios (95% confidence interval) and *P* values. The odds ratio was associated with a 1-min increase. EMT, emergency medical technician; ROSC, return of spontaneous circulation.

In the epinephrine group, the odds ratios of the time span from cardiac arrest to the first administration of epinephrine for primary and secondary outcomes, which were adjusted for the time span during which EMTs were able to administer epinephrine, are presented in Figure 3. The time span during which EMTs were able to administer epinephrine was defined as the time span from when the EMTs started CPR until ROSC or until arrival at the hospital. Early administration of epinephrine improved the neurological outcome in the epinephrine group. The adjusted odds ratio for a favorable neurological outcome was 0.917 (95% confidence interval (CI) 0.850–0.988, *P* = 0.023) per minute.

## Discussion

The present study indicated that the prehospital epinephrine administration was associated with the frequency of ROSC in the patients with witnessed out-of-hospital cardiac arrest. Furthermore, we observed that the early administration of epinephrine might improve the neurological outcome 30 days after cardiac arrest.

Recently, a few large observational studies of prehospital epinephrine administration based on the Utstein database have been reported from Japan [12–14]. Hagihara et al. indicated that epinephrine administration led to a deterioration of the neurological outcome of patients with out-of-hospital cardiac arrest [7]. However, they did not give sufficient careful consideration to the CPR time in their study [9, 15]. The other two reports indicated that early epinephrine administration may improve the neurological outcome of the cardiac arrest patients [13, 14], which was the same as the results of our study. In these two studies, in order to minimize any bias, patients who immediately had their spontaneous circulation restored without epinephrine administration were excluded from the analyses [13, 14]. In the present study, we used the propensity score, which included CPR time, in order to minimize any bias. In an observational study comparing CPR with and without epinephrine for out-of-hospital cardiac arrest, it is necessary to include both patients for whom spontaneous circulation was immediately restored without epinephrine and those patients who needed epinephrine administration. However, we should sufficiently consider the differences in the outcome between these two different types of patients.

In the two randomized control trials, administration of epinephrine did not result in an improved neurological outcome [2, 6]. In those studies, cardiac arrest patients without bystander witness were also included in the studies [2, 6]. The mean ambulance response time was 10 min in those studies, although one study did not provide the time course of CPR [2, 6]. The time spans between ambulance arrival and drug administration were not provided in their report [2, 6]. Although patients treated with late epinephrine administration were also included in these studies, epinephrine administration is likely to increase the frequency of ROSC, although it does not improve the neurological outcomes [2, 6, 16]. However, two large observational studies recently reported an improvement of the neurological outcome by early epinephrine administration [13, 14]. In the present study, the frequency of a favorable neurological outcome was found to increase 1.1 times for every 1 min earlier that the epinephrine was administered in the epinephrine group (odds ratio 0.917, 95% CI 0.850–0.988, *P* = 0.023) (Figure 3). Based on our results, it is necessary to administer epinephrine earlier in order to improve neurological outcomes in cardiac arrest patients.

Ong et al. indicated the importance of CPR time-induced bias in a study comparing CPR with and without epinephrine for out-of-hospital cardiac arrest [8]. For instance, some patients did not receive epinephrine because of early ROSC, and these patients tended to have a better neurological outcome. Some previous reports described that use of epinephrine during CPR predicted a poor outcome [7, 17–19]. However, in these reports, CPR time-induced bias was not sufficiently considered [7, 17–19]. Cardiac arrest patients with a long time to ROSC have many chances of receiving epinephrine, and these patients generally have a poorer outcome due to their longer absence of spontaneous circulation. Therefore, the patients administered epinephrine during CPR had a poorer outcome in these previous reports [7, 17–19]. To rule out the presence of any bias, a randomized double-blind placebo-control trial is usually planned. However, it is so difficult to perform a large randomized double-blind placebo-control trial about the effects of epinephrine in CPR of out-of-hospital cardiac arrest patients. In the present study, we thoroughly considered effects of CPR time. To minimize the CPR time-induced bias, we used propensity scores, which included CPR time. Furthermore, we adjusted the odds ratios of the time span from cardiac arrest to the first administration of epinephrine for the primary and secondary endpoints based on the time span during which the EMTs were able to administer epinephrine (Figure 3).

In the present study, half of the patients with out-of-hospital cardiac arrest were administered epinephrine by EMTs. However, in a large observational propensity analysis based on the Japan Utstein database, the number of patients who did not receive epinephrine administration was 25 times that of the patients who received epinephrine administration [7]. Although only qualified EMTs are allowed to administer epinephrine in Japan, one large study included patients treated by ambulance teams without qualified EMTs [7]. Furthermore, in many regions in Japan, EMTs are not permitted to administer epinephrine in patients who present with asystole as the initial cardiac rhythm in cases that are not witnessed. However, in the Sapporo trial, qualified EMTs were present in all ambulances and permitted to administer epinephrine in patients who presented with asystole as the initial cardiac rhythm. Therefore, in the present study, many patients were administered epinephrine by EMTs.

There are some limitations in the present study. Before the propensity score matching, the time span during which EMTs were able to administer epinephrine in the epinephrine group was longer than that in the no-drug group. The administration of epinephrine may be a surrogate marker of poor outcome because patients with poor outcome had many chances of epinephrine administration before the propensity score matching, such as several previous reports [7, 17–19]. In the present study, we performed the propensity analysis to balance simultaneously many covariates in the two groups and reduce the bias. However, other factors which we did not consider may be affected to outcome of the two groups in the present study. Furthermore, the competence of EMT teams may affect the relationship between epinephrine administration and patient outcomes. However, in Sapporo, all EMT teams were permitted to use epinephrine when performing CPR during the study period. In this study, the competence of the EMT teams did not differ because the study was performed at a single fire department in Sapporo. Figure 3 shows the relationship between the early administration of epinephrine and good neurological outcomes among the patients. It is clear that administering epinephrine too late does not improve the outcomes of patients with out-of-hospital cardiac arrest. However, administering epinephrine too early also does not affect the outcomes of patients. Although there is an appropriate time window for administering epinephrine during CPR, we were unable to determine this time window in the present study.

## Conclusions

Administration of epinephrine by EMTs before arrival at the hospital was associated with increase of the frequency of ROSC in patients with witnessed cardiological out-of-hospital cardiac arrest. Furthermore, the early administration of epinephrine might improve the neurological outcome of the patients with out-of-hospital cardiac arrest.

## Abbreviations

CPR: Cardiopulmonary resuscitation
EMTs: Emergency medical technicians
ROSC: Return of spontaneous circulation.
